# Correction: Dipolar vinyl sulfur fluorescent dyes. Synthesis and photophysics of sulfide, sulfoxide and sulfone based D–π–A compounds

**DOI:** 10.1039/c8ra90006j

**Published:** 2018-01-31

**Authors:** Matias Monçalves, Gabriel M. Zanotto, Josene M. Toldo, Daniel S. Rampon, Paulo H. Schneider, Paulo F. B. Gonçalves, Fabiano S. Rodembusch, Claudio C. Silveira

**Affiliations:** Departamento de Química, Universidade Federal de Santa Maria CEP 97105-900 Santa Maria-RS Brazil silveira@quimica.ufsm.br +55 55 3220-8754; Grupo de Pesquisa em Fotoquímica Orgânica Aplicada, Universidade Federal do Rio Grande do Sul – Instituto de Química Avenida Bento Gonçalves 9500. CEP 91501-970 Porto Alegre-RS Brazil fabiano.rodembusch@ufrgs.br +55 51 3308 7304 +55 51 33087204; Universidade Federal do Paraná, Departamento de Química, Laboratório de Polímeros e Catálise CEP 81531-980 Curitiba PR Brazil; Grupo de Química Teórica, Universidade Federal do Rio Grande do Sul – Instituto de Química Avenida Bento Gonçalves, 9500, CP 15003, CEP 91501-970 Porto Alegre-RS Brazil

## Abstract

Correction for ‘Dipolar vinyl sulfur fluorescent dyes. Synthesis and photophysics of sulfide, sulfoxide and sulfone based D–π–A compounds’ by Matias Monçalves *et al.*, *RSC Adv.*, 2017, **7**, 8832–8842.

The authors regret that Table 2 was incorrectly formatted in the original manuscript showing two solvent columns. The correct table, with only one solvent column is presented below.

**Table 1:** Relevant photophysical data of the fluorescence spectra, where *λ*_em_ is the emission maxima (nm), Δ*λ*_ST_ is the Stokes’ shift (nm cm^−1^), Δ*λ*_em_ is the solvatochromism in the excited state (nm cm^−1^) and *Φ*_FL_ is the fluorescence quantum yield (%)

Dye	Solvent^a^	*λ*_em_	Δ*λ*_em_	Δ*λ*_ST_	*Φ*_FL_
P1	DIO	416	36/1915	4371	0.34
	DCM	437		5365	0.22
	EtOH	434		5694	0.32
	MeCN	452		6612	0.46
P3	DIO	450	51/2262	5633	0.27
	DCM	483		6683	0.27
	EtOH	506		7634	0.45
	MeCN	501		7895	0.35
P5	DIO	450	85/3531	6533	0.29
	DCM	508		6493	0.33
	EtOH	524		7371	0.45
	MeCN	535		7975	0.20
P2	DIO	428	13/689	3590	0.26
	DCM	435		4039	0.23
	EtOH	433		4153	0.25
	MeCN	441		4498	0.25
P4	DIO	417	16/886	3643	0.44
	DCM	432		3952	0.48
	EtOH	444		4578	0.32
	MeCN	433		4683	0.16
P6	DIO	430	25/1278	3698	0.47
	DCM	449		4610	0.25
	EtOH	455		5049	0.36
	MeCN	455		5049	0.45

a DIO = 1,4-dioxane, DCM = dichloromethane, EtOH = ethanol and MeCN = acetonitrile.

The Royal Society of Chemistry apologises for these errors and any consequent inconvenience to authors and readers.

## Supplementary Material

